# Treatments of Sexual Dysfunction in Opioid Substitution Therapy Patients: A Systematic Review and Meta-Analysis

**DOI:** 10.7150/ijms.57641

**Published:** 2021-04-12

**Authors:** Fitri Fareez Ramli, Muhammad Hasif Azizi, Syed Alhafiz Syed Hashim

**Affiliations:** 1 Department of Pharmacology, Faculty of Medicine, Universiti Kebangsaan Malaysia, 56000 Cheras, Kuala Lumpur, Malaysia.; 2 Unit of Urology, Department of Surgery, Faculty of Medicine, Universiti Kebangsaan Malaysia, 56000 Cheras, Kuala Lumpur, Malaysia.

**Keywords:** bupropion, ginseng, methadone, opioids, rosa Damascena, sexual dysfunction, trazodone

## Abstract

Sexual dysfunction is a common condition in the opioid substitution therapy (OST) population. We aimed to determine the efficacy and safety of treatment for sexual dysfunction in the OST population. We searched for interventional studies from Medline, PubMed, and Scopus. Three independent authors conducted a risk-of-bias assessment (RoB 2). A total of seven studies (five randomized-controlled trials, two quasi-experimental), including 473 patients with sexual dysfunction, were identified. Among these, three bupropion (n=207), one trazodone (n=75), two rosa Damascena (n=100), and one ginseng (n=91) studies had reported significantly improve various sexual functioning domains in both genders. In a meta-analysis, bupropion significantly increased male sexual function with standardized mean difference of 0.53; 95% confidence interval of 0.19-0.88; P < 0.01; I^2^=0. The adverse effects were minor for all agents, and no significant difference between treatment and placebo groups in randomized-controlled trials. These agents have a promising future as therapy for sexual dysfunction in the OST population. However, given the limited sample size and number of studies, further studies should be conducted to confirm the use of these agents.

## Introduction

Sexual function is one of the crucial aspects of life. Although it is not a life-threatening condition, deterioration in sexual function may reduce the sexual and overall quality of life in both patients and their partners 1. Sexual problems are a broad spectrum of disorders, ranging from varying degrees of impairment of physiological components, often accompanied by psychological involvement 2. Inadequate psychophysiological response in any phase, such as desire, arousal, orgasm, or resolution, may lead to reduced libido, excitement, vaginal lubrication, or penile erection; inability to reach orgasm; overall or sexual dissatisfaction; or pain 3.

In the opioid substitution therapy (OST) population, sexual dysfunction is the commonest condition reported in numerous pharmacovigilance studies. The prevalence of sexual dysfunction varies across the population, ranging from as low as 14% to as high as 93% in men on methadone maintenance treatment (MMT) 4-8. In contrast, the data in women on MMT is limited, but the available report indicates a prevalence of 56.6% 9. Hypoactive sexual desire disorder in men and arousal impairment in women are the common sexual dysfunction in the MMT population. Moreover, the impairments are significantly higher in the MMT population compared to patients on buprenorphine maintenance treatment (BMT), another OST alternative 9, 10. On the contrary, the most common sexual dysfunctions in the general population are hypoactive sexual desire disorder and premature ejaculation in women and men, respectively 3.

Suppressive effects of the opioid on the hypothalamus-pituitary-gonadal axis are among the mechanisms of sexual dysfunction development in the OST population, particularly for potent opioid agonists such as methadone 11. The mechanism has been proven in animal studies 11. Various human studies also reported the association between hypogonadism and sexual dysfunction in patients receiving opioids 12. This mechanism is not limited to men but may also contribute to the pathophysiology of sexual dysfunction in women 13, 14. However, given the nature of the studies conducted, the established mechanism of sexual dysfunction in humans remained inconclusive 15. Moreover, sexual dysfunction in the OST population may be more complicated, as the interaction between psychological, pharmacological, and social factors may worsen the sexual dysfunction condition 6, 7, 16.

The discovery of sildenafil, a phosphodiesterase-5 (PDE5) inhibitor in 1998, has led to the development and expansion of PDE5 inhibitors class with improved pharmacological and psychological effects 17. In fact, this is the first-line therapy for erectile dysfunction (ED). Other pharmacological interventions include testosterone replacement therapy and alprostadil. The management of sexual dysfunction in men is well-established. Various international and national guidelines are available to guide the clinician in managing patients with sexual dysfunction. These guidelines are not limited to ED but include other sexual dysfunctions, such as premature ejaculation 18-20. The advancement of sexual dysfunction management in women occur in later years but has received wide attention over the past decade 21. Similarly, some guidelines are available to guide various female sexual dysfunctions management, such as low desire, low arousal, orgasmic dysfunction, and sexual pain 21.

In contrast, the management of sexual dysfunction in the OST population is still limited and not widely studied. Lack of standardized management of sexual dysfunction in the OST population might be attributed to the lack of awareness and knowledge regarding sexual dysfunction among OST patients 6, 11. A study conducted in methadone clinics that evaluated ED-related health-seeking behaviour reported almost half of the patients who were aware of having ED did not seek any treatment. Moreover, seeking a physician for help was the least popular treatment option in those who sought the treatment 6.

Due to the difference in terms of the pathophysiology of sexual dysfunction, the treatment option of sexual dysfunction in the OST population is different from the other population. Antidepressants such as bupropion and trazodone and herbal alternatives such as rosa Damascena and ginseng have been reported to significantly improve OST patients with sexual dysfunction 8, 22-27. Although various clinical studies and reviews have explored the role of intervention for sexual dysfunction in many conditions, such as diabetes, stroke, and cancer population, the effectiveness and safety of sexual dysfunction intervention in the OST population has not been studied thoroughly 28-30. We aimed to evaluate the effectiveness and safety profile of the treatments for sexual dysfunction from interventional studies in the OST population.

## Materials and Methods

We adhered to the Preferred Reporting Items for Systematic Reviews and Meta-Analyses (PRISMA) guideline in preparing the review and article, except we did not register our protocol in any database 31.

### Literature Search

**Figure 1:**
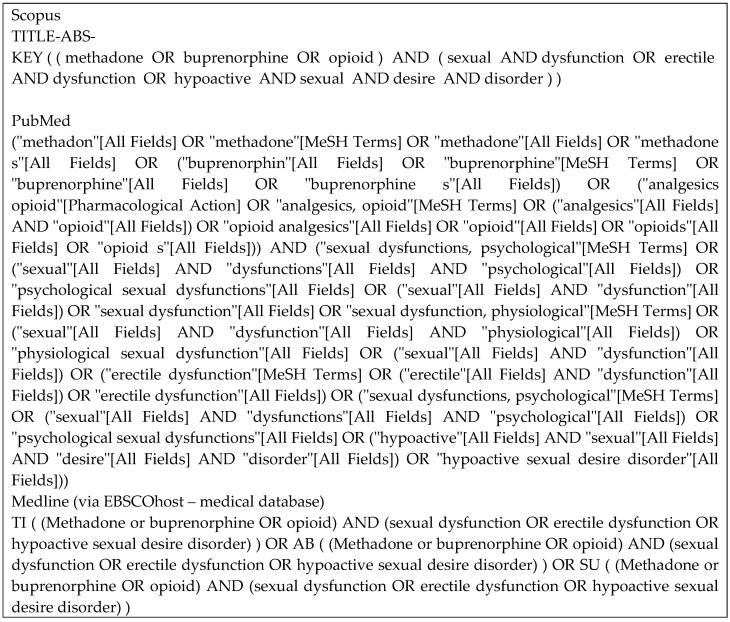
Keywords strategies for each database.

Articles from three databases, Medline (via EBSCOhost - Medical Database), Scopus, and PubMed, were searched using keywords of (methadone or buprenorphine OR opioid) AND (sexual dysfunction OR erectile dysfunction OR hypoactive sexual desire disorder) from inception until November 2020 by two investigators (F.F.R. and S.A.S.H.) independently and any disagreement was resolved by discussion (Figure 1). Title screening was initially done, followed by abstract and full-text screening based on inclusion and exclusion criteria.

### Eligibility Criteria

#### Inclusion Criteria

- Study designs: All interventional trials, including randomized-controlled trials (RCTs) and quasi-experimental;
- Participants: Studies in male and female patients aged ≥18 years with sexual dysfunction of any severity on opioid substitution therapy (methadone or buprenorphine) for opioid use disorder;
- Interventions: Any pharmacological intervention, including herbal preparation as treatment. The comparator of active intention included placebo or other active pharmacological intervention;
- Outcomes: Studies with any mean score of validated questionnaires for sexual function assessment such as Arizona Sexual Experience Scales (ASEX) 25, Brief Sexual Function Inventory 22, 23, Clinical Global Impression for Sexual Function (CGI-SF) 27, Erectile Dysfunction Intensity Scale 8, 26, Female Sexual Function Index 22, 24, International Index of Erectile Function with 15-item 22, 23, 27, and Sexual Desire Inventory -2 27. Any minor and major adverse effects related to the interventions and the number of participants who dropped out and adverse effects were also retrieved;

- Data accessibility: Studies that were published as full papers and in English.

#### Exclusion Criteria

- Study designs: Any observational, animal study, and review articles;
- Participants: Patients on an opioid for pain management and sexual dysfunction secondary to organic causes;
- Interventions: Non-pharmacological intervention such as sex therapy or switching to another OST;
- Data accessibility: Studies published in languages other than English.

### Data Extraction

The data from the included articles were extracted; first author's name, year of publication, country, population, number of participants, study design, age, gender, method (inclusion and exclusion criteria), duration and dose of OST, intervention (type, dose, route of administration, frequency, duration), and outcomes (sexual function and adverse effects). Any disagreement about the eligibility of the studies was resolved by discussion. For feasibility reason, authors were not contacted for missing data.

### Risk-of-bias Assessment

The risk-of-bias assessment was conducted by three independent investigators (F.F.R., S.A.S.H., M.H.A.) using a revised tool for risk-of-bias (RoB 2) assessment for RCTs. Any disagreement was resolved by discussions 32. Briefly, RoB 2 provides a risk-of-bias assessment in five domains; randomization process, deviation from the intended interventions, missing outcome data, measurement of the outcome, and selection of reported results 32. Assessors are guided by signalling questions and domain-specific algorithms. There are three possible judgements by the authors that include “low risk of bias”, “some concerns”, or “high risk of bias” 32.

### Data Synthesis and Analysis

Treatment effects were measured if there were at least two included studies to compare. The treatment effects were calculated using standardized mean differences (SMD) with a 95% confidence interval (CI), given the difference in terms of tools used to measure sexual function. Forest plot inspection, standard chi-square (p-value=0.1), and I^2^ statistics were used to assess heterogeneity of meta-analysis in the case of no substantial clinical and methodological heterogeneity detected 33-35. The *p*-value and I^2^ statistics determined the effects model used. Our study utilized the random-effects model as heterogeneity existed in terms of doses and the treatment's duration. We used Review Manager (RevMan) [Mac], Version 5.4, the Cochrane Collaboration, 2020.

## Results

### Description of paper selection process

**Figure 2:**
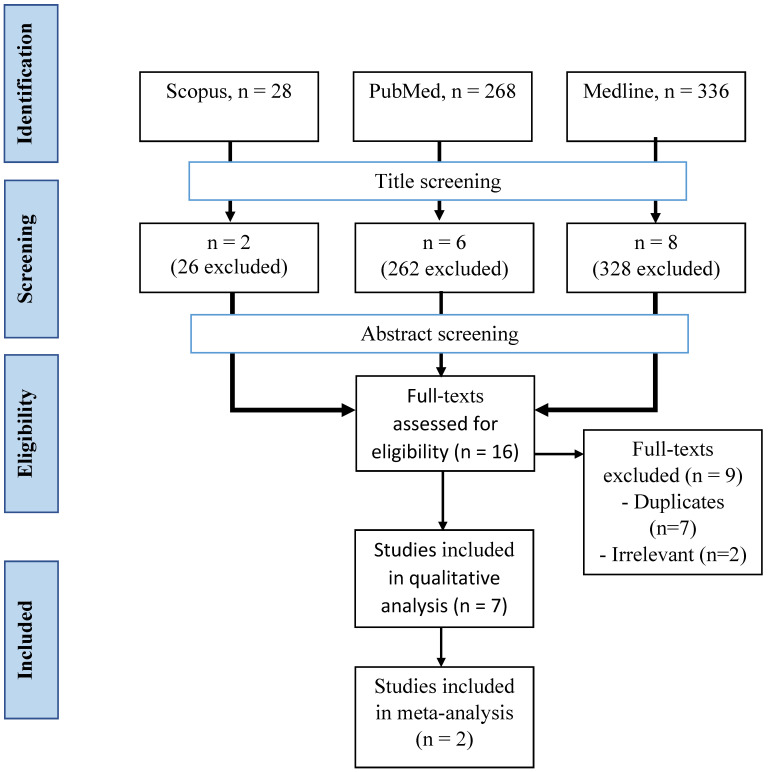
Selection process of the articles.

A total of 632 articles were retrieved from three databases (Figure 2). After the screening of the title, 616 articles were removed, leaving 16 articles behind. All articles from the three databases were merged, and nine articles were removed. A final seven articles were included in our study after screening based on abstracts and full texts.

### Characteristics of included studies

The majority of the studies were conducted in Iran, and only one study was conducted in Malaysia (Table 1). All studies were conducted in the MMT population with a sample size range of 50-91. Five studies were RCTs and two quasi-experimental studies. Most of the study population consisted of the male population. Only two studies had reported treatment for sexual dysfunction in the female population. All RCTs required the MMT duration of at least six months, and sexual dysfunction developed after MMT treatment. Two quasi-experimental studies had set the minimum duration of MMT at 30 days 8, 26. Other eligibility criteria included lack of the following conditions; other psychiatric comorbidities or conditions 10, 22-25, other substance use disorder 23, 24, 36, obvious organic illnesses 8, 26, 27, 36, not on other psychotropic drugs 8, 22-24, 27, 36, anti-viral treatment 8, 24, 27, and sexual enhancement therapy 8, 24, 27, somatic problems 22, current marital issues 22, non-adherence to the protocol 24, allergy 22, intention to become pregnant, pregnancy, or lactation 24, and adverse effects related to treatment 24.

### Treatments for sexual dysfunction

Three studies conducted using bupropion with a dose of 100-300 mg for 6-8 weeks (Table 2). Two studies conducted using Rosa Damascena in the male and female population. Another study used ginseng or trazodone.

### Risk-of-bias assessment

Risk-of-bias assessment for all RCT studies demonstrated all, but one had a low risk of bias for the randomization process (Table 3). Only Salehi, Barekatain 36 had an unclear risk of bias for the randomization process domain. Four out of five studies had a high risk of bias in the selection of the reported result domains.

### The effects of treatment on sexual function

The majority of the studies had reported improvement in a variety of sexual function domains (Table 4). Both rosa Damascena and ginseng were reported to improve sexual drive, erection, ejaculation, problem assessment, sexual satisfaction, and overall sexual function in the male MMT population. On the other hand, bupropion was reported to improve erectile function, sexual desire, dyadic sexual desire, intercourse satisfaction, and overall sexual function in one RCT study. In contrast, another study reported a trend towards sexual function improvement but non-significant results in terms of overall sexual function.

In the female MMT population, both ginseng and rosa Damascena were reported to improve desire, arousal, orgasm, pain, and overall sexual function (Table 5). However, the mixed results were reported in terms of lubrication and satisfaction between these two treatments.

#### Meta-analysis of bupropion effect on sexual function

**Figure 3:**
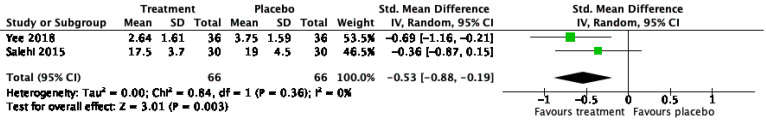
Forest plot on effect of bupropion on male sexual dysfunction.

Only two RCT studies on bupropion as a treatment of sexual dysfunction were eligible for meta-analysis. The mean and standard deviation of the total ASEX score 36 and CGI-SF 27 were included in the analysis to calculate SMD. A meta-analysis of two RCT studies with a total of 132 patients demonstrated a significant effect of bupropion in the treatment of sexual dysfunction with a SMD of 0.53; 95% confidence interval of 0.19-0.88; P < 0.01; I^2^=0 (Figure 3).

### Dropout rate and adverse effects of the treatments

The total dropout rate ranged from 0-36.3% in four studies. The reasons for dropout included no reason, adverse reactions (AEs), no sexual partner, not effective, and other reasons. No studies reported any major AE. Only mild AEs were reported, including insomnia, inability to concentrate, and rashes in patients receiving bupropion. One-third of patients receiving trazodone had reported sedation. In patients receiving ginseng, AEs included agitation, sleeplessness, high blood pressure, stomach ache, diarrhoea, and rash. No AEs were reported and mentioned in patients receiving rosa Damascena.

## Discussion

Our systematic review found four potential pharmacological agents; bupropion, trazodone, rosa Damascena, and ginseng 8, 22-27. Most of the studies were conducted in the male MMT population 8, 22, 23, 25-27. The male predominance might be attributed to a low number of studies in the female MMT population and a higher proportion of men in this population 8, 22, 23, 25-27. In risk-of-bias assessments for RCTs, most of the studies had high risk-of-bias in reporting 22, 23, 25, 27. The majority of these studies had not specified certain measurement tools in the registered protocol and selective outcome measurement and analysis 22, 23, 25, 27.

We were unable to proceed with meta-analysis for trazodone 26 and ginseng 22 because only one study was found in our systematic review. In terms of rosa Damascena, two studies included were not homogenous as one was conducted in the male MMT population, while another in the female MMT population. Given the differences in gender and sexual functioning, we did not proceed with meta-analysis. Furthermore, another reason is the quasi-experimental nature of trazodone 26 and bupropion 8 studies. In contrast, we found two studies on bupropion that were eligible for further analysis 25, 27.

We found that bupropion significantly improved overall sexual functioning, sexual desire, erectile function, and intercourse satisfaction in men 8, 27, 36. Both quasi-experimental and RCT had reported positive impacts in numerous domains (sexual desire, erectile function, intercourse satisfaction) of sexual function. However, the effects of bupropion on other domains were not significantly improved (orgasmic function and overall satisfaction). Moreover, our meta-analysis had reported significant positive effects of bupropion on male sexual function. Our results agree with another RCT study conducted in the male population on selective serotonin reuptake inhibitor (SSRI) treatment for depression 37. The underlying mechanism of bupropion is related to its non-typical mechanism of action via inhibition of noradrenaline and dopamine reuptake without compromising serotonin regulation. Serotonin enhancement is related to sexual dysfunction in patients on SSRIs 38. The dual-action of bupropion on depression and sexual dysfunction at a higher therapeutic dose has the advantage for the use in the MMT population, in which at least half of the population is affected by depression 39. Other than that, increased testosterone is another potential mechanism of improved sexual function in MMT patients on bupropion treatment 27.

In terms of adverse reaction, no major or significant difference was found between treatment (bupropion) and placebo groups 8, 27, 36. Yee, Loh 27 reported that insomnia was the highest adverse effect, affecting approximately one-tenth of the study population. Similarly, Safarinejad 37 reported that no major AEs occurred in patients with SSRI-induced sexual dysfunction patients. However, minor AEs related to neurological, musculoskeletal, respiratory, and gastrointestinal were reported to be significantly higher in patients receiving bupropion 37. These AEs might be attributed to the pharmacological effect of bupropion on the enhancement of the functions and blockades of dopamine and noradrenaline receptors 38.

Trazodone is another potential pharmacological agent for the treatment of sexual dysfunction in men. Tatari, Farnia 26 had conducted a study to determine the effects of trazodone in 75 MMT patients with ED. Despite a significant improvement, the report is limited in terms of which specific domain of erectile function was improved. A meta-analysis study of trazodone effects on erectile function reported a significant improvement in patients with psychogenic cause, but not for physiological and mixed-related ED 40. Interestingly, significant improvement in the MMT population was achieved at a low dose (50 mg/day) 26, a dose that exerts hypnotic and neuroprotective effects and not anti-depressant effect 41. The mechanism of action of trazodone is attributed to its high affinity and blockade of alpha-1 adrenoceptor in penile tissue 42. Concerning adverse reaction, sedation was the only adverse effect reported, leading to dropping out of the patients 26. A similar finding was reported in a systematic review conducted by Fink, MacDonald 40 in the general population.

Ginseng is an herbal alternative for the treatment of sexual function. Farnia, Alikhani 22 had reported significant improvement in numerous domains of sexual function in both genders. Similar effects were reported in other populations such as men and postmenopausal women 43, 44. Sexual improvement by ginseng might be explained by the local and central effects of ginsenosides, the active components present in ginseng. Ginsenoside may cause vasodilation and relaxation of the corpus cavernosum, facilitating penile erection directly or via nitric oxide released from perivascular nerves or endothelial cells 45. Moreover, ginseng causes sexual improvement via neurohormonal effects at hypothalamus and pituitary levels 45. The mechanism of ginseng in women might be attributed to the ability of ginseng to enhance estrogen levels, promoting estrogenic changes on the female reproductive system, such as vaginal smooth muscle relaxation and increased vaginal blood flow 46, 47. Adverse effects of ginseng reported were related to minor neurological, rash, and gastrointestinal symptoms. Similar findings were reported in other populations 44. Other than that, Oh, Chae 44 reported vaginal bleeding in postmenopausal women treated with ginseng.

Another herbal medicine reported to have the potential for sexual dysfunction in the OST population is Rosa Damascena. Two studies had reported significant improvement in male and female patients on MMT. The underlying mechanism is not fully understood. Rosa Damascena's effects on the hypothalamus-pituitary-gonadal axis may partly explain its sexual enhancement effects. Farnia, Tatari 24 reported significant elevations of estradiol and progesterone and reduction of prolactin in females receiving rosa Damascena. In the male MMT population, serum testosterone levels were significantly increased in the treatment group 23. Similar findings were reported in male and female patients with SSRI-induced sexual dysfunction in the major depressive disorder 48, 49. The preclinical study supports the positive effect in elevations of follicle-stimulating hormone, luteinizing hormone, and testosterone in male rats treated with rosa Damascena 50. However, negligible correlations were observed between hormonal levels and sexual function in the male and female MMT population 23, 24.

Despite promising evidence of treatment for opioid-induced sexual dysfunction, these findings should be interpreted with caution due to the limited number of studies, small sample size, and strict exclusion criteria. Six out of seven studies were conducted in Iran. We postulated a high prevalence of patients with opioid misuse and the availability of natural resource that is native to Iran (Rosa Damascena) are the reasons why most of the published data on sexual treatment mainly came from Iran. In contrast, other regions in the world may have different treatment strategies, such as switching methadone with buprenorphine, an agent with a lower risk of sexual dysfunction 10. These factors may limit the generalizability of the studies to other populations. Sexual dysfunction in OST patients is complex. Most of the studies in our report included opioid use disorder patients only on methadone without any comorbidity or the use of other prescribed or illicit drugs. Many patients on OST have a dual diagnosis or other comorbidities in the real-world situation, which requires other pharmacological intervention. Methadone has wide pharmacokinetic interindividual variability, which may be affected by drug-drug interaction, genetic mark-up and underlying disease 16. Another limitation is the lack of adverse effects reporting in three RCTs, making it difficult to assess safety profile in meta-analysis. The result must be interpreted with caution, given the high risk-of-bias in most of the studies secondary to selective outcome reporting. Future research should emphasize on a larger sample size, more studies in other countries, adherence to registered protocol, and improved quality of reported articles to reduce the risk of bias. Recommendations for quality improvement would include an explanation for protocol deviation and a statement regarding the safety profile or adverse effect, even if no adverse effects were observed during the trials.

## Conclusions

Bupropion, ginseng, rosa Damascena and trazodone have a promising future as therapy for male sexual dysfunction, particularly in sexual desire, erection, ejaculation, problem assessment, and sexual satisfaction domains. In the female OST population, ginseng and rosa Damascena may have the potential to treat sexual dysfunction related to desire, arousal, lubrication, orgasm, satisfaction, and pain. However, given the limited sample size and number of studies, further studies should be conducted to confirm the efficacy and safety.

## Figures and Tables

**Table 1:** Characteristics of included studies

Author's name	Year	Country	Study population	n	Design	Gender (%)	Age (years)
Tatari 26	2010	Iran	MMT	75	Quasi	Men (100%)	39.5^†^ (18-51^‡^)
Tatari 8	2014	Iran	MMT	67	Quasi	Men (100%)	36.2^†^ (21-53^‡^)
Salehi 25	2015	Iran	MMT	60	RCT	Men (100%)	P:43.1^†^; T: 41.5^†^)
Farnia 23	2017	Iran	MMT	50	RCT	Men (100%)	40.0^†^
Farnia 24	2017	Iran	MMT	50	RCT	Women (100%)	38.8^†^
Yee 27	2018	Malaysia	MMT	80	RCT	Men (100%)	42.8^†^ (25-60^‡^)
Farnia 22	2019	Iran	MMT	91	RCT	Men (64.9%), Women (35.1%)	W: 39.0^†^; M: 40.6^†^

^†^mean; ^‡^range. Abbreviations: M: men; MMT: methadone maintenance treatment; n: sample size; NA: not available; P: placebo group; RCT: randomized-controlled trial; T: treatment group; W: women.

**Table 2:** Types, route, dose and duration of treatment of sexual dysfunction in MMT population

Ref	Agent	Route	Dose daily	Duration (week)
26	Trazodone	Oral	100 mg	6
8	Bupropion	Oral	100 mg	6
25	Bupropion	Oral	200 mg	8
23	Rosa Damascena	Oral	2ml	8
24	Rosa Damascena	Oral	2ml	8
27	Bupropion	Oral	300 mg	6
22	Ginseng	Oral*	1000 mg	4

*Each capsule contained 250 mg dry powder of ginseng radix. Abbreviations: MMT: methadone maintenance treatment; Ref: reference.

**Table 3:** Risk-of-bias assessment for included randomized-controlled trials

Reference	Randomization process	Deviations from intended intervention	Missing outcome data	Measurement of the outcome	Selection of the reported results	Overall risk
25	?	+	+	+	-	-
23	+	+	+	+	-	-
24	+	+	+	+	+	+
27	+	+	+	+	-	-
22	+	+	-	+	-	-

+ low risk-of-bias; ? some concern; - high risk-of-bias.

**Table 4:** Effects of treatment on male sexual functioning in the MMT population

Tools		ASEX	BSFI						CGI-SF	EDIS	IIEF						SDI-2		
Ref	Agent	Total	Sexual drive	Erection	Ejaculation	Problem assessment	Sexual satisfaction	Total	Total	Total	Erectile	Orgasmic	SD	Intercourse satisfaction	Overall satisfaction	Total	DSD	SSD	Total
26	Trazodone									↑*									
8	Bupropion									↑*									
25	Bupropion	NS																	
23	Rosa Damascena		↑^‡^	↑^‡^	↑^‡^	↑^‡^	↑*^‡^	↑^‡^								↑^‡^			
27	Bupropion								↑*^†^		↑*^†^	NS	↑^†^	↑*	NS	↑^†^	↑*^†^	NS	↑^†^
22	Ginseng		↑^‡^	↑^‡^	↑^‡^	↑^‡^	↑*^‡^	↑^‡^								↑*^‡^			

*Treatment vs placebo (at the end of the treatment); ^†^within treatment group (baseline vs week 2/4/6); ^‡^Time x Group interaction. ASEX: Arizona sexual experience scale; BSFI: Brief Sexual Function Inventory; CGI-SF: Clinical Global Impression Scale adapted for Sexual Function; DSD: dyadic sexual desire; EDIS: erectile dysfunction intensity scale; EF: erectile functioning; IIEF: International Index of Erectile Function; MMT: methadone maintenance treatment; SD: sexual desire; SDI: Sexual desire inventory -2 (Malay version); SF: sexual function; SSD: solitary sexual desire.

**Table 5:** Effects of treatment on female sexual functioning in the MMT population

Tools	FSFI						
Ref	Desire	Arousal	Lubrication	Orgasm	Satisfaction	Pain	Total
24	↑*^‡^	↑*^‡^	↑^‡^	↑^‡^	↑^‡^	↑^‡^	↑*^‡^
22	↑^‡^	↑^‡^	NS	↑*^‡^	NS	↑^‡^	↑^‡^

FSFI: Female Sexual Function Inventory; MMT: methadone maintenance treatment; NS: not significant; *Treatment vs placebo; ^†^within treatment group (baseline vs week 2/4/6); ^‡^Time x Group interaction; ↑: improved.

**Table 6:** Dropout rate, reason and adverse effects of patients on treatment of sexual dysfunction

Ref	n	Dropout			Reason	Adverse effects
		Tx	Placebo	Total		
26	75	15	-	15 (20%)	33.3% due to sedation.	Sedation
8	67	15	-	15 (22.4%)	Not specified	No major side effects.
25	60	Nil	Nil	Nil	Nil	Not reported
23	50	Nil	Nil	Nil	Nil	Not reported
24	50	Nil	Nil	Nil	Nil	Not reported
27	80	14	15	29 (36.3%)	No reason: 4 vs 5^†^ AE: 9 vs 5^†^ No sexual partner: 1 vs 1^†^ Not effective: 0 vs 3^†^ VA: 0 vs 1^†^ EPTB: 1 vs 0^†^	No significant different between groups. AEs in treatment groups: insomnia, skin itchiness, cannot concentrate, constipation.
22	M:54	4	2	6 (11.1%)	AE: 4 vs 0^†^; NC: 0 vs 4^†^	Sleeplessness, agitation, high BP, stomach ache, diarrhoea, rash.
	W:37	6	5	11 (29.7%)	AE: 5 vs 3^†^; NC: 1 vs 2^†^	

^†^Treatment vs placebo; AE: adverse effect; BP: blood pressure; EPTB: extrapulmonary tuberculosis; -: no control group; W: women; M: men; NC: non-compliance; Ref: reference; Tx: treatment; VA: vehicle accident.

## Abbreviations

AE: adverse effects
ASEX: Arizona Sexual Experience Scales
CGI-SF: Clinical Global Impression for Sexual Function
CI: confidence interval
ED: erectile dysfunction
MMT: methadone maintenance treatment
OST: opioid substitution therapy
PDE-5: phosphodiesterase-5
PRISMA: Preferred Reporting Items for Systematic Reviews and Meta-Analyses
RCT: randomized-controlled trial
RoB2: revised tool for risk of bias
SMD: standardized mean differences
SSRI: selective serotonin reuptake
